# Involution of classic Kaposi sarcoma lesions under acitretin treatment Kaposi sarcoma treated with acitretin

**DOI:** 10.1002/ccr3.3428

**Published:** 2020-10-22

**Authors:** Najla Daadaa, Asmahane Souissi, Meryam Chaabani, Ines Chelly, Moez Ben Salem, Mourad Mokni

**Affiliations:** ^1^ Department of Dermatology La Rabta Hospital Tunis Tunisia; ^2^ Department of Pathology La Rabta Hospital Tunis Tunisia; ^3^ Medical office Tunis Tunisia

**Keywords:** acitretin, generalized pustular psoriasis, Kaposi sarcoma

## Abstract

Acitretin, indicated for generalized pustular psoriasis, was effective in concomitant classic Kaposi sarcoma.

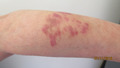

## 1 INTRODUCTION

Acitretin, indicated for generalized pustular psoriasis, was effective in concomitant classic Kaposi sarcoma.

Kaposi sarcoma is a multicentric angioproliferative spindle cell disease of lymphatic endothelial origin. We report a 60‐year‐old woman with Kaposi sarcoma successfully treated with systemic retinoid prescribed for a generalized pustular psoriasis.

Kaposi sarcoma (KS) is a rare, multicentric angioproliferative spindle cell disease of lymphatic endothelial origin.^1^ Systemic treatments currently used for KS are liposomal anthracyclines, paclitaxel, other cytotoxic agents (vinblastine, vincristine, bleomycin), or interferon‐α.^2^

We report a 60‐year‐old woman with KS successfully treated with systemic retinoid prescribed for a generalized pustular psoriasis (GPP).

## 2 CASE PRESENTATION

**FIGURE 1:**
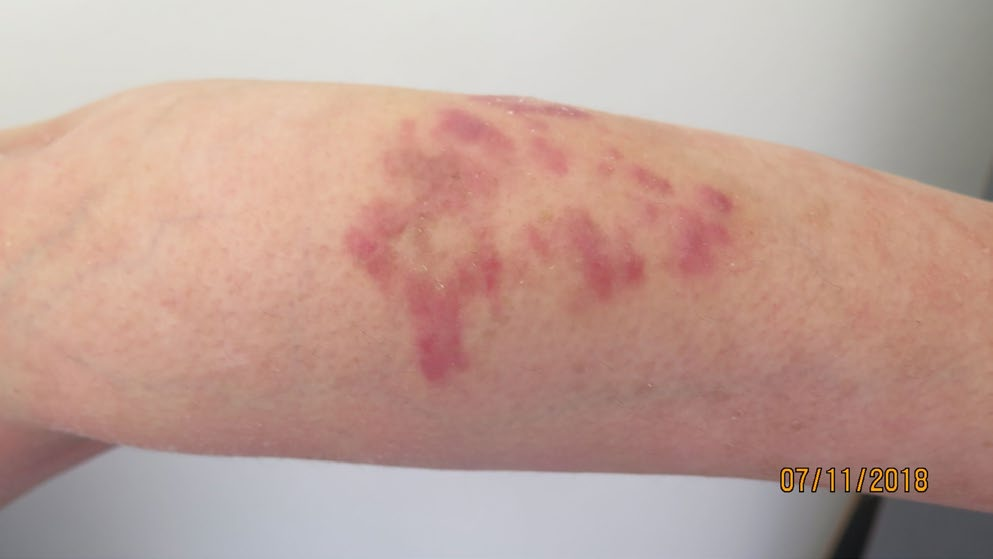
Angiomatous infiltrated papules and plaques on the left arm

**FIGURE 2:**
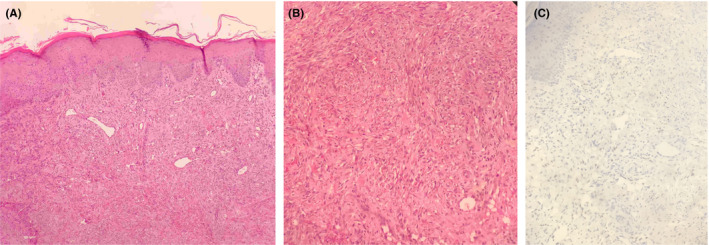
Kaposi sarcoma histology. A, Newly formed cleft‐like vascular spaces with diffuse dermal neoplastic proliferation (hematoxylin and eosin, original magnification ×200). B, Proliferation of spindle‐shaped cells in the dermis (hematoxylin and eosin, original magnification ×400). C, Many nuclei immunoreactive for human papillomavirus‐8 (HHV‐8 immunohistochemistry stain, ×400)

**FIGURE 3:**
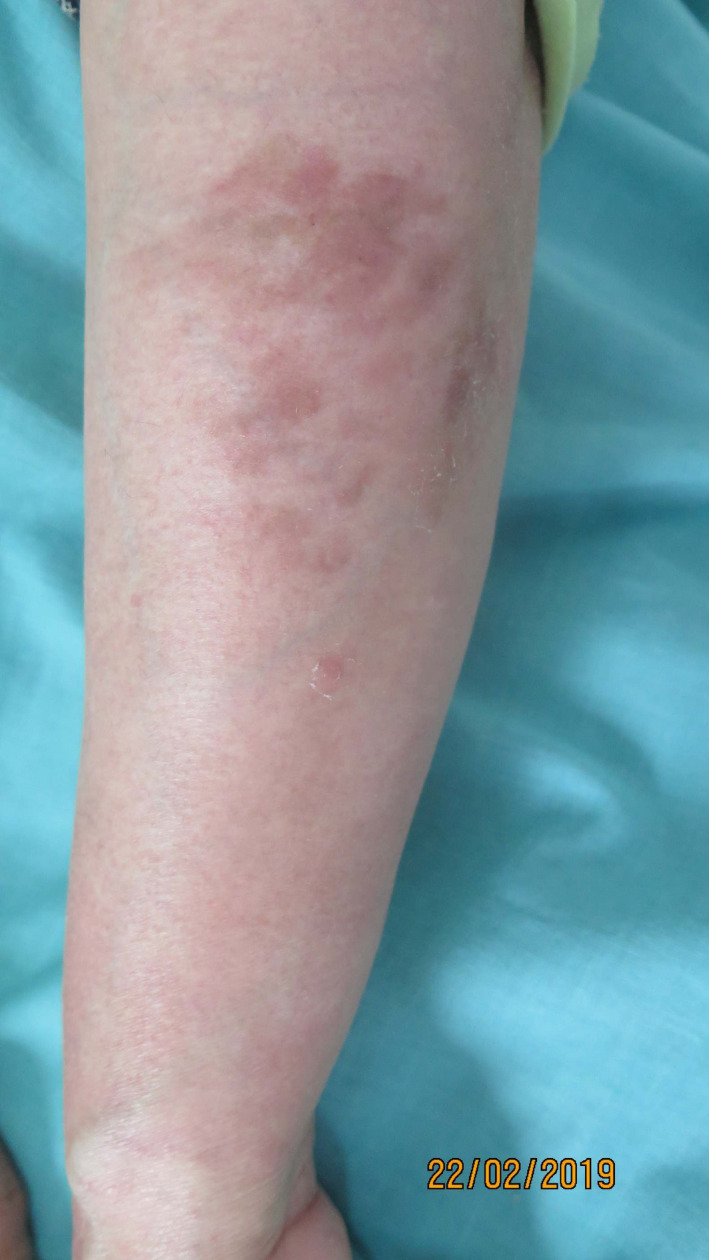
Regression of the angiomatous papules and plaques on the left arm

A 60‐year‐old woman without any past medical history was admitted to our department for a progressively developing pustulosis during the last 15 days. Physical examination showed high‐grade fever, malaise, and a generalized eruption with a painful erythema and coalescent 2‐3 mm pustules. Blood count revealed neutrophilia (30 000 elements/mm^3^). C‐reactive protein was elevated with a value of 173 mg/L. The procalcitonin test was negative. Histology showed spongiform pustules in the stratum corneum surrounded by parakeratosis and psoriasiform hyperplasia. The diagnosis of GPP was established. The patient was put on acitretin 30 mg daily (0.4 mg/kg) with an excellent improvement of both general and skin conditions. After 15 days of hospitalization, a few, red‐brown to violaceous, infiltrated papules and plaques appeared on her both forearms (Figure 1). There was no mucosal involvement. A skin biopsy revealed the presence of newly formed cleft‐like vascular spaces and diffuses dermal spindle cell neoplastic proliferation (Figure 2A,B). Immunohistochemistry showed the presence of HHV‐8 confirming the diagnosis of KS (Figure 2C). Tests for HIV were negative. Three months later, while the patient was only under acitretin treatment, we noticed the complete regression of angiomatous plaques of KS (Figure 3).

## 3 DISCUSSION

In our patient, although without major evidence, acitretin appears to be effective in a newly established KS.

Previous reports highlight the safeness of retinoid therapy in KS, mainly in the AIDS‐related variant.^3^, ^4^, ^5^, ^6^ Retinoids have been also described as an effective treatment of classic KS. Florek reported stabilization of KS with 35 mg oral acitretin daily in a 77‐year‐old man while tretinoin, imiquimod, and alitretinoin 0.1% gel twice daily did not result in significant improvement.^1^

Morganroth had treated a classic KS with topical 0.1% alitretinoin gel in an 83‐year‐old woman. Remission of almost all the lesions was obtained after a few months with only mild irritative dermatitis.^7^ Recently, topical alitretinoin (9‐cis‐retinoic acid) has been approved by the US Food and Drug Administration and European consensus‐based interdisciplinary guideline as a treatment for the localized KS.^2^, ^8^

The Table 1 summarizes the reports in which the efficacy of retinoids in KS had been assessed.

**Table 1:** Different reports assessing the efficacity of retinoids in Kaposi Sarcoma

	Number of patients	Age (y)	Sex	Type of KS	Molecule	Dose	Outcomes	Duration of treatment	Side effects
Bower et al 1997	15	39	15 men	AIDS‐related KS	Oral isotretinoin	1 mg/kg 2 daily doses	Improvement (7%) Stable disease (38%)	At least 4 wk	Cheilitis, skin dryness, xerophthalmia, nausea, arthritis, leukopenia anemia, hepatic cytolysis
Saiag et al 1998	19	35	19 men	AIDS‐related KS	Oral tretinoin	45 mg/m^2^ daily	Improvement (14 patients)	12 wk	Cheilitis, transient headaches, skin dryness, dry mucoses, Gingivitis, ototoxicity, nausea, vomiting, Influenza‐like illness
Bodsworth et al 2001	62	38	62 men	AIDS‐related KS	Alitretinoin gel 0.1% (9‐cis retinoic acid)	Twice daily	Improvement (37%)	12 wk	Rash, pruritus, parasthesia, pain, peeling
Miles et al 2002	60	37.5	58 men/2 women	AIDS‐related KS	Oral alitretinoin	Once daily oral doses of 100 mg/m^2^	Improvement (37%)	15 wk (median duration)	Headache, skin toxicity, depression, increased triglyceride and cholesterol levels, pancreatitis
Morganroth 2002	1	83	Woman	Classic KS	Alitretinoin gel 0.1%	Twice daily	Remission	6 mo	Irritative dermatitis
González de Arriba et al 2007	1	83	Woman	Immunosuppression‐related KS	Alitretinoin gel 0.1%	Twice daily	Remission	5 mo	Local erythema, edema, and blistering
Florek et al 2015	1	77	Man	Classic KS	Oral acitretin	35 mg daily	Stabilization of KS	Nonmentioned	Nonmentioned

Pharmacologically, it has been proved that retinoic acid and its synthetic analogs inhibit the proliferation of KS cells by down‐regulation of IL‐6 (an autocrine growth factor for KS cells). Their effects are exerted through two families of nuclear receptors, retinoic acid receptors, (RARs) and retinoid X receptors (RXRs), which belong to the superfamily of steroid‐thyroid‐vitamin D3 hormone receptors. These receptors antagonize the enhancer action of NF‐IL6, a basic zipper family transcription factor and, therefore, inhibit IL‐6 promoter action.^9^

Corbeil has demonstrated that acitretin at low concentrations was sufficient to inhibit the growth of rapidly dividing early passage KS cells. Higher concentrations induced KS cells apoptosis but they are pharmacologically unachievable with oral systemic therapy.^10^ Retinoic acid analogs also inhibit HHV8 replication.^11^

The coexistence of psoriasis and KS is rarely reported in the literature.^9^ In a large study conducted by Brambilla et al, of the 1407 patients followed for KS, 37 patients were identified with psoriasis. All of these patients had psoriasis vulgaris, and none of them had GPP. The average time latency time from psoriasis onset to KS onset was 18.8 years.^12^ An intense cytokine dysregulation during GPP may explain the acceleration of the development of HHV‐8 infection into KS in our case.

In summary, we would like to highlight that acitretin, prescribed for GPP in our patient, was an active antitumor drug for early classic KS with complete recovery within a few weeks. Our observation is also particular given the short latency time from GPP to KS onset. The efficacy of acitretin is interesting to assess in KS.

## CONFLICT OF INTEREST

None declared.

## AUTHORS' CONTRIBUTION

ND and AS: managed the patient and drafted the manuscript. IC: analyzed all histopathology specimens. MBS: referred the patient. MM: managed the patient and revised the manuscript. All authors have approved the final manuscript.

## ETHICAL APPROVAL

Appropriate consent has been obtained, prior to submission, for the publication of images and data.

## Data Availability

Data sharing was not applicable to this article as no datasets were generated or analyzed during the current study.
